# Theory of Mind Deficits in Bipolar Disorder in Remission

**DOI:** 10.1192/bjo.2022.178

**Published:** 2022-06-20

**Authors:** Shravani Chauhan

**Affiliations:** Roseberry Park Hospital, Middlesbrough, United Kingdom

## Abstract

**Aims:**

Theory of mind (ToM) is the ability to represent one's own and other's mental state. Studies in bipolar affective disorder show mixed results possible due to confounding factors like intelligence, attention, phase of illness and current mood. Purpose of this study is to study ToM in remittent bipolar disorder patients and compare with normal controls to find if there are residual deficits during remission

**Methods:**

40 bipolar patients in remission and 40 age and sex matched controls were recruited. Clinical remission for 3 months with YMRS <4 and HAM-D <7 was inclusion criteria. ToM was assessed by Faux Pas test. Data were analysed using SPSS-11.5 for Windows with parametric and non-parametric tests as indicated. Level of significance taken as p < 0.05 (two tailed).

**Results:**

Mean age of onset of illness in patient group was 23.8 years with duration of illness 11.3 years. Mean number of episodes 6.7 and duration of remission 4.15 months. ToM test result revealed deficit in recognizing social cues in faux pas test by bipolar patients as compared to normal controls. There was no difference between both groups in test result on control stories.

**Conclusion:**

Results suggest that ToM deficits are present in bipolar disorder patients even during apparent clinical remission, indicating it may be a trait marker of the illness. There is no deficit in understanding a regular social context without faux pas. It also revealed that there is no correlation with ToM and duration of illness

